# Studies on the Effect of Oxidation on Bioactivity of Phenolics and Wine Lees Extracts

**DOI:** 10.3390/antiox12040931

**Published:** 2023-04-14

**Authors:** Zhijing Ye, Jinlin Shi, Roland Harrison, Richard Hider, Alaa El-Din A. Bekhit

**Affiliations:** 1 School of Viticulture and Wine Science, Te Pūkenga, Trading as Eastern Institute of Technology, Napier 4112, New Zealand; 2 Department of Wine, Food and Molecular Biosciences, Lincoln University, Lincoln 7647, New Zealand; 3 Department of Food Science, The University of Otago, Dunedin 9054, New Zealand

**Keywords:** wine lees, phenolic compounds, antioxidant, antimicrobial activity, oxidation

## Abstract

It is expected that any processing and handling of lees (e.g., drying, storage or removal of residual alcohol using various concentration techniques) will expose the material to oxidation and the consequences of oxidation on the biological activity of the lees and the lees extracts are unknown. The effects of oxidation (using horseradish peroxidase and hydrogen peroxide model system) on phenolic composition and their antioxidant and antimicrobial activities were investigated in (i) a flavonoid model system composed of catechin and grape seed tannin (Cat:GST) extracts at various ratios and (ii) in Pinot noir (PN) and Riesling (RL) wine lees samples. For the flavonoid model, oxidation had a minor or no impact on total phenol content but increased (*p* < 0.05) total tannin content from approximately 145 to 1200 µg epicatechin equivalent/mL. An opposite observation was found in the PN lees samples where oxidation reduced (*p* < 0.05) the total phenol content (TPC) by approximately 10 mg GAE/g dry matter (DM) lees. The mean degree of the polymerization (mDP) values of the oxidized flavonoid model samples ranged from 15 to 30. The Cat:GST ratio and interaction of the Cat:GST ratio with oxidation were found significantly to affect the mDP values of the flavonoid model samples (*p* < 0.05). Oxidation increased the mDP values in all oxidized flavonoid model samples except for Cat:GST 0:100. The mDP values of the PN lees samples ranged from 7 to 11 and remained the same after oxidation. There was no significant reduction in the antioxidant activities (DPPH and ORAC) of the model and wine lees after oxidation except the PN1 lees sample (decreased from 3.5 to 2.8 mg Trolox equivalent/g DM extracts). In addition, no correlation was observed between mDP (approximately 10 to 30) and DPPH (0.09) and ORAC assay (−0.22), which indicates that the higher mDP resulted in a poor ability to scavenge DPPH· and AAPH· free radicals. Antimicrobial activities of the flavonoid model were found to be improved after the oxidation treatment against *S. aureus* and *E. coli* with minimum inhibition concentration (MIC) of 1.56 and 0.39 mg/mL. This may indicate that new compounds were formed during the oxidation treatment, and these compounds showed more effective microbicidal activity. LC-MS work is required in the future to identify the compounds that are newly formed during the oxidation of the lees.

## 1. Introduction

The utilization of phenolic compounds from wine wastes as a source of biologically active compounds has attracted the attention of many researchers focusing on grape seeds, grape skins and pomace [1,2,3,4,5,6]. For example, flavonoids are attracting great interest due to their good antioxidant, antimicrobial, antiviral and antifungal activities and their potential health benefits. Flavan-3-ols are a major class of flavonoids. In recent years, flavan-3-ols in different plant sources have been found to exhibit strong biological activities [1,7,8,9]. Gris et al. [10] found that the flavan-3-ol content was positively correlated with the antioxidant activity of Brazilian *Vitis vinifera* red wine. Flavan-3-ols in wine exist as monomers and polymers, and also exist polymerized with other flavonoids and form diverse compounds [11]. Flavan-3-ols are abundant in wine by-products [2]. The majority of studies that investigated wine wastes focus on grape pomace, skin and seeds [1,2,3,4,5,6], and little attention has been paid to the wine lees [12,13]. Ye et al. [12,13] characterized wine lees from different vinification backgrounds and reported that flavan-3-ols were the primary flavonoids in the PN lees; and gallic acid (0.5–2.2 mg/g DM extract) was the predominant non-flavonoid. The concentrations of catechin, epicatechin and total procyanidins in the PN lees were 0.4 to 8.0 mg/g DM extract, 0.9–5.8 mg/g DM extract, and 1.4–4.8 mg/g DM extract, respectively. To support the use of the lees commercially, different methods to allow the storage of the lees need to be devised. It is expected that any processing and handling of the lees (e.g., drying, storage or removal of residual alcohol using heat or other economical means) will expose the material to oxidation and the consequences of such actions on the biological activity of the lees and the lees extracts are unknown. To gain insights on this gap of knowledge, a series of experiments were conducted using a lees model containing catechin and grape seed tannin at various ratios and subjected to oxidation using hydrogen peroxide and horseradish peroxidase. A comparable investigation of the effects of oxidation on the biological activities of the PN and Riesling (RL) wine lees was conducted. In all instances, total phenol content (TPC), total tannin content (TTC), mean degree of polymerization (mDP) and antioxidant and antimicrobial activities of the treated samples were determined.

## 2. Materials and Methods

### 2.1. Chemicals

Sodium carbonate, anhydrous di-sodium hydrogen orthophosphate, and ascorbic acid were from BDH (Poole, UK). Methylcellulose, gallic acid, phloroglucinol, resveratrol, and hydrogen peroxide were purchased from Sigma Chemical Co. (St. Louis, MO, USA). Trolox (6-hydroxy-2,5,7,8-tetramethylchroman-2-carboxylic acid), Folin–Ciocalteu reagent, α,α-diphenyl-β-picrylhydrazyl (DPPH), 2,2′-azobis (2-amidinopropane) dihydrochloride (AAPH), catechin, epicatechin, epigallocatechin, epigallocatechin gallate, horseradish peroxidase (140 U/mg), malvidin, caftaric acid, cinnamic acid, resveratrol, quercetin methyl-glucoside and quercetin were obtained from Sigma-Aldrich Chemical Co. (Steinheim, Germany). Hydroxybenzoic acid was purchased from Fisons Scientific Apparatus (London, UK). Grape seed tannin (GST) was obtained from Tarac technologies, (North Adelaide, Australia). Sodium dihydrogen phosphate monohydrate was purchased from LabServ Biolab (Clayton, Australia). Ethanol (100%) and charcoal were obtained from Fisher Scientific (Loughborough, UK). Methanol (100%), acetic acid and hydrochloric acid were obtained from Merck (Darmstadt, Germany). Ammonium metavanadate was purchased from Acros Organics (Morris Plains, NJ, USA). Silver nitrate was from UNIVAR (Auckland, New Zealand). Trichloroacetic acid was from BDH (Poole, England). Anthocyanin standards used for the LC-MS analysis (i.e., peonidin-3-glucoside, petunidin-3-glucoside, delphinidin chloride, cyanin chloride, cyanidin chloride, pelargonidin chloride and malvidin chloride) were purchased from Extrasynthése (Genay, France). All reagents and chemicals used in this study were of analytical grade or higher.

### 2.2. Wine Lees Sample Preparation

#### 2.2.1. Preparation of Flavonoid Model

The model system prepared for this oxidation trial was catechin and grape seed tannin (GST), and the design was modified from the methodology reported by Jongberg et al. [14] for the oxidation of myosin. To make the flavonoid model, mixtures of catechin and GST were mixed according to Table 1 (with total weight of 200 mg) and then were dissolved in 2.4 mL ethanol and the volume was made up to 20 mL with DI water. A 19.2 mL aliquot of 260 mM TRIS buffer (pH 7.5) was added to the mixture and the pH was adjusted using TRIS buffer as the activity of HRP reach maximum at pH 7.5 following the product information. The total volume of the flavonoid model was 39.2 mL. The flavonoid model system was stored in a tightly closed container in the dark at 4 °C. The flavonoid model were mixed with 10.8 mL of aqueous horseradish peroxidase (50 U) in the oxidation assay mixture (total volume for the treatment was 50 mL) as described below.

#### 2.2.2. Wine Lees

Four fresh lees samples were kindly provided by Pegasus Bay Winery, Waipara, New Zealand. Triplicate samples of the lees were from Pinot noir (PN1 and PN2) and Riesling (RL1 and RL2). The information of the collected fresh lees was demonstrated in Appendix A. Fresh lees were used for the oxidation treatment.

#### 2.2.3. Oxidation of Flavonoid Model and Real Lees Samples

The experiment design is shown in Appendix B. The flavonoid model system was oxidized by hydrogen peroxide (H_2_O_2_) in the presence of horseradish peroxidase (HRP) [14]. To prepare a HRP stock (1500 U), 0.0107 g of HRP was dissolved in 600 µL deionized water. The HRP stock was aliquoted (30 µL) into Eppendorf tubes and stored at −20 °C until use. Hydrogen peroxide (0.5 M) was prepared from a stock solution of 10 M H_2_O_2_. To initiate the oxidation, 1.8 mL 0.5 M H_2_O_2_, 20 µL HRP (50U) and 8.98 mL of deionized water were added to the prepared flavonoid model (39.2 mL, pH = 7.5). The total volume of this model system was 50 mL, and the reaction was carried out in centrifuge tubes. The mixture was vortexed and left at room temperature for 60 min. For the control, 1.8 mL 0.5 M H_2_O_2_ and the 20 µL HRP (50 U) were replaced by deionized water. At the end of 60 min, 10 mL of control and oxidized samples were taken and individually were mixed with 10 mL of methanol (100% analytic grade) to re-extract the phenolics.

For the oxidation of the fresh lees samples, 38.46 g of PN1 (13.0% DM), 61.35 g of PN2 (8.2% DM), 133.87 g of RL1 (22.4% DM) and 191 g of RL2 (15.7% DM), were weighed and then topped up to 200 g by adding 12% ethanol followed by adding 192 mL 260 mM TRIS buffer (pH 7.5). The reason for weighing the wine lees above was to ensure there was enough wine lees and phenolic compounds for sample extraction and chemical analyses [13]. Preliminary studies indicated that the oxidation of the lees was found to be optimal when HRP was 500 U. To initiate the oxidation, 18 mL of 0.5 M H_2_O_2_, 200 µL HRP (500 U) and 89.8 mL of deionized water were added to the wine lees. The total volume of the lees system was 500 mL. For the control, 0.5 M H_2_O_2_ was replaced by 18 mL deionized water. The reaction was carried out in Schott bottles. The mixture was mixed and left at room temperature for 24 h. At the end of the oxidation time, the wine lees extracts were extracted as described by Ye et al. [13] and Mercurio et al. [15]. The freeze-dried lees samples (Virtis Freezemobile 12SL, New York, NY, USA; at a pressure of 0.5 mbar) underwent a two-step extraction using acidified 50% ethanol (*v*/*v* containing 1% *v*/*v* HCl) (Table 2). The sample was shaken using a thermostatic orbital shaker at 150 rpm for 1 h at 20 °C (Model OM 11, Ratek Instrument Ltd., Boronia, VIC, Australia). Samples were then centrifuged at 2890· *g* for 5 min using a Megafuge 1.0 with a Siehe rotor (Kendo Laboratory Products, Hanau, Germany). The extraction process was repeated. The supernatants from both extractions were combined, aliquoted and stored at −20 °C for subsequent chemical analyses.

The oxidized lees were extracted with acidified 50% ethanol and extracts were kept at −20 °C for chemical analysis.

### 2.3. Determination of Total Phenol Content (TPC)

The total phenol content of the wine lees extracts was determined using method as described by Singleton et al. [16]. The absorbance of the mixture was read at 765 nm using a visible spectrophotometer (UNICAM Helios Alpha, Cambridge, UK) against a water blank. The analysis was performed with triplicate measurements of three samples for each extract. The concentration of total phenolic was calculated from a standard curve constructed using gallic acid standard. The range of gallic acid standard concentration was between 0 mg/L and 1000 mg/L. The results were expressed as mg of gallic acid equivalent per liter (mg GAE/L).

### 2.4. Determination of Total Tannin Content (TTC)

The total tannin content of the freeze-dried wine lees extracts was determined using the methylcellulose precipitable (MCP) tannin assay as described by Sarneckis et al. [17]. This is the Australian Wine Research Institute (AWRI) standard method for total tannin content measurement in grape homogenates and red wines. The assay total volume was 1 mL and the absorbance of the mixture was read using an UV spectrophotometer at the absorbance of 280 nm. Epicatechin was used to construct a standard curve in the range of 0, 25, 50, 75 and 100 μg/mL and the tannin content was reported on epicatechin equivalent basis (μg epicatechin equivalent/mL).

### 2.5. Determination of Phenolic Profile

The quantification of individual phenolic compounds was determined using the method reported by Kemp et al. [18]. The flavonoid model and wine lees extracts were filtered using a 0.45 μm PTFE filter (2165 Catalogue, Grace Davison) before injection. The filtered samples were injected (10 μL) into an LC-MS system (Shimadzu 2010, Kyoto, Japan) equipped with a PDA detector (D2 and W Lamp 200–600 nm) and a Grace Davison C18 column (250 × 2.1, particle size 5 μm, held at 25 °C). The flow rate was kept at 0.5 mL/min throughout running time. The analysis was carried out with electrospray ionization (ESI) interface, and the MS parameters were a detector voltage of 1.5 kV, an interface voltage of 4.5 kV, a curved desolvation line of −45 V, an array reflector voltage of 150 V, and heat block and CDL temperatures that were kept at 200 and 250 °C, respectively. Phenolic compounds in the samples were confirmed individually by mass spectroscopy and were quantified with a PDA detector at 280 nm using corresponding standards.

### 2.6. Determination of the Mean Degree of Polymerization (mDP)

The mean degree of polymerization (mDP) of the wine lees extracts was determined as described by Kennedy and Jones [19]. The method is based on the acid-catalyzed cleavage of proanthocyanidin in the presence of excess phloroglucinol followed by chromatographic analysis of the reaction products. The lees samples were prepared according to Ye et al. [13]. The freeze-dried lees was extracted using 50% ethanol (*v*/*v*) followed by centrifugation at 3000× *g* for 5 min using a Megafuge 1.0 with a Siehe rotor (Kendro Laboratory Products, Hamburg, Germany). Supernatants were decanted and stored at −20 °C. An Agilent HPLC 1100 series was used to analyse phloroglucinol adducts using a Phenomenex Luna C18 (5 µm, 250 × 4.60 mm) column protected by a C18 column guard. The column temperature was 25 °C. Two mobile phases (A and B, containing 2% *v*/*v* aqueous acetic acid and 2% *v*/*v* acetic acid in methanol, respectively) were used and the eluting peaks were monitored at 280 nm. The flow rate was set at 0.8 mL/min, with the mobile phase set initially as 95% mobile phase A/5% mobile phase B for 5 min, followed by 90% mobile phase A/10% mobile phase B for 25 min, then 60% mobile phase A/40% mobile phase B for 30 s, and finally, 100% mobile phase B for 6 min. Catechin and epicatechin were used as standards in the range from 0 to 100 ppm. The molar sum of all flavan-3-ol monomers and phloroglucinol adducts was divided by the molar sum of all flavan-3-ol monomers to calculate mDP [11].

### 2.7. Determination of Antioxidant Activities

The antioxidant activity of the wine lees extracts were determined using the α,α-diphenyl-β-picrylhydrazyl radical (DPPH·) and Oxygen Radical Antioxidant Capacity (ORAC) methods.

The ability of phenolic extracts to scavenge the DPPH· was evaluated as previously described by Brand-Williams et al. [20] and Sánchez-Moreno et al. [21] with the modifications described by Ye et al. [13]. The results were also presented in EC_50_ (the amount of sample required to decrease the initial concentration of DPPH· by 50%). The EC_50_ of different extracts was calculated from the regression equation obtained from plotting the percentage of DPPH· vs. extract concentration.

The antioxidant capacity of the extracts was determined using ORAC assay according to as described by Ye et al. [13]. FLUOstar Omega multifunctional microplate reader (BMG LABTECH, Ortenberg, Germany) was used to measure the fluorescein intensity. The instrument was set up to read fluorescence intensity mode. The excitation, emission and cut off wavelength were 485 nm, 538 nm and 530 nm, respectively, and the gain was adjusted to 85%. The measurement was taken over 60 min at 1 min interval. All determinations were performed on three subsamples and the measurements were in triplicate. The range of Trolox standard concentration was between 0 mg/mL and 0.05 mg/mL. The results were expressed in equivalent Trolox concentration (mg TE/mL).

### 2.8. Determination of Antimicrobial Activity

The antibacterial and antifungal activity of the extracts were determined by conducting the minimum inhibitory concentration (MIC) assay in microtiter plates with nutrient broth. Bacteria (Gram positive *Staphylococcus aureus*, Gram negative. *Escherichia coli*) and fungi (*Candida albicans*) were incubated in Mueller–Hinton broth (MHB) and Sabouraud Dextrose broth (SAB). Assay conditions were those described by Wood and Washington [22]. The MIC values were measured by a microplate reader (BioTek^®^ Synergy 2, Gen 5^TM^, Winooski, VT, USA) after 24 h of incubation of the mixture at 37 °C for *E. coli* and *S. aureus* and 48 h for *C. albicans*. Absorbance readings using the microplate reader were set at 600 nm at 37 °C. All measurements of the MIC_50_ values were performed in triplicate, including three controls with 50% acidified ethanol, MHB and microorganisms.

### 2.9. Statistical Analysis

Analysis of variance (ANOVA) was carried out to investigate the effects of samples (dependent variable) on the measured parameters. Significant differences at *p*-value < 0.05 were determined using Tukey’s multiple comparisons test. Error bars in graphs indicate the standard deviation (SD) of the mean. Pearson correlation coefficients, among the measured parameters, were also calculated. Genstat^®^ software (Version 9.0, VSNI, Hemel Hempstead, UK) was used for statistical analysis.

## 3. Results and Discussion

### 3.1. Effect of Oxidation on Total Phenolic Content (TPC) and Total Tannin Content (TTC)

**Figure 1:**
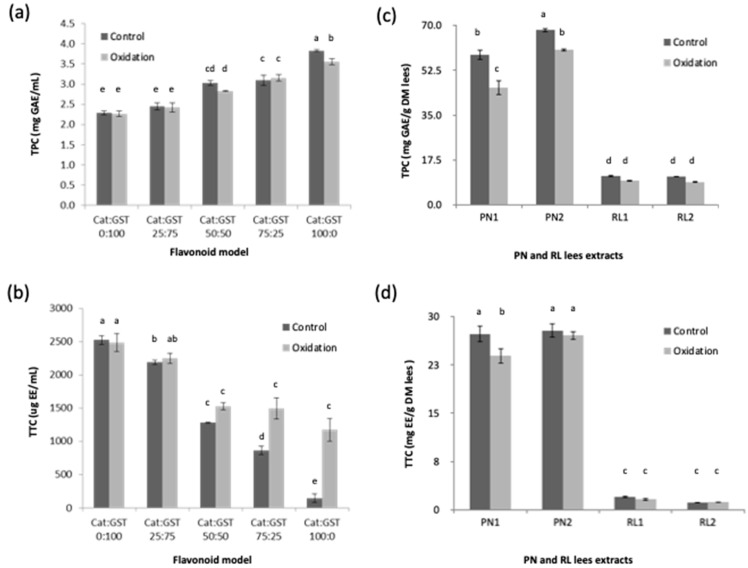
Total phenol content (TPC, mg GAE/mL) and total tannin content (mg EE/mL) of flavonoid model (**a**,**b**) and Pinot noir (PN) and Riesling (RL) wine lees extracts (**c**,**d**) exposed to oxidation with H_2_O_2_ and horseradish peroxidase (oxidized) and not exposed to oxidation (control). Means that do not share a letter (a–e) are significantly different (*p* < 0.05). Error bars are standard deviation of replicate subsamples samples (n = 3) and measured in triplicate. GAE: gallic acid equivalent; EE: epicatechin equivalent.

The TPC values of the different samples of the flavonoid model (Cat:GST mixtures) were determined, and the results are expressed as mg GAE/mL (Figure 1a). The lowest and highest TPC values were observed in Cat:GST 0:100 (Control: 2.29 ± 0.05 and Oxidized: 2.27 ± 0.07 mg GAE/mL) and Cat:GST 100:0 (Control: 3.83 ± 0.03 and Oxidized: 3.55 ± 0.08 mg GAE/mL), respectively. The Cat:GST mixtures were expected to have same TPC values before and after oxidation. The composition of the flavonoid model mixture has a significantly impact on Folin–Ciocalteu assay results (*p* < 0.05) where increasing the ratio of catechin in the flavonoid model mixture increased the TPC value of the sample. Oxidation had no significant effects on TPC except for Cat:GST 100:0 treatment where the TPC of the oxidized samples was lower than the control (*p* < 0.05). In general, the TPC values of the different flavonoid models (control and oxidized) suggested that oxidation may have no significant impact on TPC except when catechins are the only chemical component available in the reaction mixture.

The TPC of both the control and oxidized lees extract samples are shown in Figure 1b. As expected, the highest TPC values were observed in the PN lees samples while the lowest TPC values were found in the RL lees samples (*p* < 0.05) [13]. For the PN lees extract samples, oxidation had a significant impact on TPC (*p* < 0.05). For example, the mean TPC values of the control PN1 and PN2 were 58.4 ± 2.0 and 68.2 ± 0.8 mg GAE/g DM lees, but after oxidation, the TPC values of the PN1 and PN2 samples were decreased significantly to 45.7 ± 2.9 and 60.4 ± 0.6 mg GAE/g DM lees, respectively. Low TPC values were found in the RL wine lees samples both pre- and post-oxidation (ranged from 9.0 ± 0.3 to 11.4 ± 0.4 mg GAE/g lees). Similarly, the TPC values were mathematically lower in the oxidized RL lees than the control ones, but no significant differences were found between the RL lees samples (*p* > 0.05).

Because the control and oxidized versions of Cat:GST mixtures and lees samples did not always have the same TPC, this suggested that some of the newly formed compounds cannot be detected by the Folin–Ciocalteu assay. The total phenol content by Folin–Ciocalteu assay is based on the measurement of the total hydroxyl concentration of phenolic compounds [23]; the oxidation of phenolic compounds causes loss of the hydroxyl group.

The TTC results of the flavonoid model are expressed as µg epicatechin equivalent (EE)/mL (Figure 1c). For the flavonoid model, the lowest and highest TTC values were observed in the control Cat:GST 100:0 (145.08 ± 65.15 µg EE/mL) and Cat:GST 0:100 (control, 2523.10 ± 65.02 and oxidized, 2484.10 ± 135.76 µg EE/mL). A trend was observed where the TTC values of the control samples were decreased with increasing of catechin in the Cat:GST ratio. This is attributed to the fact that methyl cellulose used in the tannin assay reacts with polymeric tannin. Thus, catechins (monomeric flavan-3-ols) in the control samples cannot interact with methyl cellulose. The TTC values were not different when the catechin concentration in the mixture was ≤50% (*p* > 0.05). Significant differences were found between the TTC values of the control and oxidized samples that have Cat:GST at a ratio of 75:25 and 100:0 (*p* < 0.05). This indicated that new polymer compounds were generated by the oxidation. Nagarajan et al. [24] reported water-soluble oligo (epicatechin) were formed in a hydrogen peroxide dependent oxidation reaction. Hosny and Rosazza [25] reported three different biphenyl C-C dimers were formed in this oxidation reaction. In the samples with a GST ≥ 50% ratio, there were no differences between the TTC values of the control and oxidized samples (*p* > 0.05). However, other reactions may still be occurring with no contribution to the TTC results, e.g., the intramolecular reactions within the same molecule may take place during oxidation. Zanchi et al. [26] suggested that aromatic rings are connected with kernels of polymeric tannin via covalent bonds as a result of oxidation, which lead to more rigid structures.

The TTC results of the flavonoid model are expressed as mg epicatechin equivalent (EE)/g DM lees (Figure 1d). The TTC values of the PN lees (ranged from 23.9 ± 1.2 to 26.7 ± 0.3 mg EE/mL) were higher (*p* < 0.05) than the RL lees (ranged from 1.1 ± 0.1 to 2.0 ± 0.2 mg EE/mL), which is consistent with the findings reported by Ye et al. [13]. There were no significant differences between the TTC values of the control and oxidized wine lees samples. The TTC value of PN1 was reduced from 27.31 ± 1.29 to 23.89 ± 1.22 mg EE/mL after oxidation. This is consistent with the flavonoid model that the catechin concentration in the mixture was ≤50% (Cat:GST 0:100, 25:75, and 50:50).

In general, the TTC values of the different flavonoid model systems (control and oxidized) indicated that oxidation and the generation of polymerized tannins can have a significant impact when catechin is present at ≥50% of the phenolics ratio. At lower GST concentrations, catechin becomes a target for the free radicals generated from H_2_O_2_ and HRP and polymerizes leading to increased TTC. In comparison, the changes in the TTC values of the wine lees with/without oxidation were similar to those found in the Cat:GST 0:100 samples. This might indicate that the phenolics in the wine lees were mostly polymeric tannins. To support this contention, the phenolic composition and mean degree of polymerization of the lees samples were investigated.

### 3.2. Effect of Oxidation on Phenolic Compounds and mDP

#### 3.2.1. Phenolic Compounds

The identification of phenolic compounds in the flavonoid model and wine lees both pre- and post-oxidation is shown in Table 3 and Table 4.

Non-flavonoids and flavonoids compounds were identified in both the control and oxidized flavonoid model samples (Table 3). Gallic acid was the major non-flavonoid found in the flavonoid model sample when GST was present in the mixture (Cat:GST 0:100, 25:75, 50:50 and 75:25) and its content ranged from 1.7 ± 0.9 to 11.9 ± 0.1 ppm. Other non-flavonoids including caftaric acid, p-coumaric acid and hydroxybenzoic acid were also identified in the flavonoid model and the amounts of these organic acids were generally less than 5 ppm. Catechin was the major flavonoid identified with a content ranging from 189.4 ± 7.2 to 1879.4 ± 124.3 ppm. Other flavonoids including epicatechin and procyanidins B and C were also found and their content decreased with the decreasing proportion of GST, which suggested GST was the origin of these compounds.

After oxidation, the content of gallic acid, catechin, epicatechin and procyanidins B and C was decreased in comparison with the non-treated control samples. For example, the content of gallic acid remaining post-oxidation decreased to 8.9 ± 3.5 and 1.7 ± 0.3 in Cat:GST 0:100 and Cat:GST 50:50, respectively; and was not present in Cat:GST 75:25. Similar observations were also found with procyanidins B and C where both dimers were not present in oxidized Cat:GST 25:75. Catechin was reduced dramatically and the percentage of catechin remaining was about 45% in the flavonoid model with a mixing ratio of Cat:GST 100:0. Previous studies have provided evidence that the new compounds generated from phenolic compounds during enzymatic oxidation include the formation of epicatechin gallate trimer and tetramer [27,28] and adibenzotropolone A [29]. These observations indicated that monomeric and dimeric phenolic compounds undergo modifications that lead to the generation of new compounds or tannins. The newly formed compounds can be dimers, e.g., the content of procyanidin A was increased in oxidized samples.

Major procyanidins identified in the flavonoid model with high proportion of catechin were catechin dimers with C4-C8 or C4-C6 bonding. In addition, the contents of catechin remaining in the oxidized Cat:GST 100:0 samples were found to be lower than the control samples even after taking into consideration the formation of procyanidin. Nagarajan et al. [24] carried out a similar enzymatic (HRP) oxidation study with epicatechin and reported the presence of water-soluble oligomeric epicatechins. The apparent loss of catechin may therefore be attributed to the formation of oligomeric catechins that were not detected by HPLC method used in the present study.

Table 4 demonstrates non-flavonoids and flavonoids that were identified in the PN and RL lees. Among the control samples, gallic acid was the major non-flavonoid found in the PN sample in the range from 1.64 ± 0.01 to 2.03 ± 0.01 mg/g DM extract. Other non-flavonoids such as caftaric acid, p-coumaric acid and hydroxybenzoric acid were not identified in the PN samples. Catechin was the major flavonoid that was identified at a concentration range from 3.63 ± 0.09 to 3.80 ± 1.50 mg/g DM extracts. Other flavonoids including epicatechin, procyanidin B and C were all at lower concentrations than the catechin. Similar trends were observed in the RL samples, except for epicatechin that was the major flavonoid (2.43 ± 0.14 mg epicatechin/g extract) found in RL2 sample.

After oxidation, the content of gallic acid, catechin, epicatechin and procyanidin B was decreased compared to the control samples. For example, the content of gallic acid in the PN samples post-oxidation decreased from 1.64 ± 0.01 and 2.03 ± 0.01 to 0.07 ± 0.01 and 0.43 ± 0.18 mg gallic acid/g DM extracts, respectively, and was almost absent in the RL samples. Catechin and epicatechin contents in PN samples (Table 4) were reduced dramatically with only 3% and 20% remaining, respectively.

Procyanidin A slightly increased in PN2 only after oxidation, which is consistent with the results found in the flavonoid model (Table 3). Procyanidin B in the PN samples was not affected by oxidation and only the RL2 sample decreased by oxidation in (Table 4) where the compound was present. In addition, a significant increase in procyanidin C was observed in the PN samples (*p* < 0.05) that ranged from 3.88 ± 0.23 to 4.22 ± 0.10 mg catechin/g DM extracts, while the concentration of catechin, epicatechin and epicatechin gallate decreased. The increase in procyanidin C might be due to the formation of dimeric catechin [25], epicatechin gallate trimer and tetramer [14,15] in oxidation. Further analysis with LC-MS would be required to identify the composition of the procyanidin formed in oxidation.

#### 3.2.2. Mean Degree of Polymerization (mDP)

**Figure 2:**
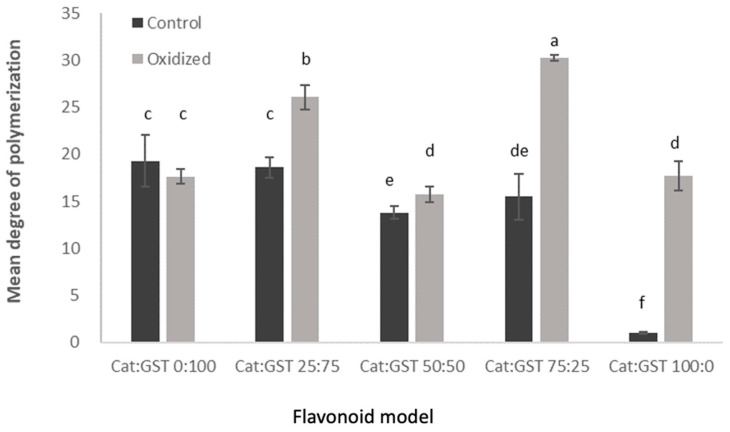
Mean degree of polymerization (mDP) of flavonoid model. Means that do not share a letter (a–f) are significantly different (*p* < 0.05). Error bars are standard deviation of replicate subsamples samples (n = 3) and measured in triplicate.

The mean degree of polymerization is a parameter that can be used to define the average number of monomeric units in a polymer or oligomer molecule. It is also an important parameter for the characterization of proanthocyanidin in wine. Figure 2 shows the mean degree of polymerization of both the control and oxidized flavonoid model and the lees samples. Among the control samples, GST was the major contributor to the mDP value of the flavonoid model because the catechin monomers should be removed during the washing of the Sep-Pac C18 cartridge (prior to acid-hydrolysis step). Theoretically, the mDP values of the control Cat:GST 0:100 and 25:75, 50:50 and 75:25 should be the same. Cat:GST 0:100 and 25:75 possessed the highest mDP (approximately 19), followed by Cat:GST 50:50 and 75:25, which had mDP values of 13.8 and 15.5, respectively. This is attributed to increasing monomeric catechin in the calculation of the average of degree of the polymerization values because of inadequate removal of the catechin monomers; in fact, the range of values was rather similar, from 14 to 19.

The mDP values of the oxidized flavonoid model samples ranged from 15 to 30, which is greater than the range of the mDP values of the control sample (14–19). The Cat:GST ratio and interaction of the Cat:GST ratio with oxidation were found significantly to affect the mDP values of the flavonoid model samples (*p* < 0.05). Oxidation increased the mDP values in all oxidized samples except Cat:GST 0:100 (Figure 2). However, this does not mean that there were no structural changes during oxidation because the mDP values only show the average of all polymers in the analyzed sample. Vernhet et al. [30] characterized oxidized tannin (at 0.1 to 10 g/L) in deionized water (the pH was adjusted to 3.5 using trifluoracetic acid) and reported intramolecular reaction generated polymers with different conformation depending on the initial tannin concentration. Polymers were found in the oxidized Cat:GST 100:0 sample with a mDP value of 17. This is different from the findings of [25], which demonstrated the formation of three different dimeric (+)-catechin products after oxidation in the presence of hydrogen peroxide and horseradish peroxidase. This might be attributed to the formation of biaryl and A-type linkages in the oxidized Cat:GST 100:0 sample, which was stable during the acid hydrolysis process of the mDP analysis [30,31], and up to 80 to 90% of some wine tannin in the experimental wine cannot be deploymerized due to their complexity [32]. Therefore, the mDP values of Cat:GST 25:75, 50:50 and 75:25 may only represent the polymer that can be depolymerized and bind with phluroglucinol.

The mean degree of polymerization (mDP) of the wine lees (Table 5) PN1 and PN2 was 10.7, and 6.7, respectively. Ye et al. [12] determined the mDP of the phenolic extractd obtained from Pinot noir and Pinot noir rosé wine lees. The mDP values of PN 1 and PN2 lees in the present study are within the range (8.7 to 12.7) reported for various Pinot noir wine lees. The mDP of PN1 and PN2 are lower than the Pinot noir rosé lees (mDP = 34.6) [12]. This can be attributed to the higher concentration of anthocyanin in PN than Pinot noir rosé. The mDP of the wine lees are not signifiantly different pre- and post-oxidation, being 10.96 ± 0.51 and 10.60 ± 0.90 in PN1, and 6.72 ± 0.36 and 6.77 ± 1.19 in PN2, respectively. In red winemaking, grape skin and seeds are normally left in the must prior to fermentation and during the fermentation process, which allows greater tannin extraction, where for rosé, there is a limited or no skin contact in winemaking. Polymeric proanthocyanidin can be generated during the wine aging as a result of oxidation. The size of the polymers in wine can be limited by the presence of anthocyanin due to the anthocyanin-tannin interaction [33].

The mDP values of both the control and oxidized RL lees samples were not able to be determined due the low amount of depolymerized tannin for mDP determination. Vernhet et al. [30] reported the formation of new structures in oxidized apple and grape seed tannin fractions that were partly resistant to acid-catalyzed cleavage and possibly left dimer and/or trimer in that system that could affect the analytical accuracy of the method.

The composition (%) of each monomer of both terminal and extension units in the breakdown of polymeric proanthocyanidin in PN is shown in Table 5. Among the control samples, catechin (about 58% and 64%) was the predominant terminal units of polymeric proanthocyanidin extracted from the PN lees; epicatechin was also a major terminal unit of polymers in the samples; epicatechin was the predominant extension unit of the PN lees (58% and 59%). The finding is consistent with the previous study [12]. Ye et al. [12] characterized the composition of polymers isolated from five different PN lees. The predominant terminal and extension units were catechin (56–61%) and epicatechin (62–75%). After oxidation, the predominant terminal and extension units remained the same. In the current study, the percentage of catechin and epigallocatechin in the extension units of the PN lees samples increased from 7% and 31% to 12% and 44%, respectively. This might suggest that catechin and epigallocatechin were involved in polymerization during the oxidation. This explained the reduction in monomeric catechin in Table 4.

### 3.3. Effect of Oxidation on Antioxidant Activities

**Figure 3:**
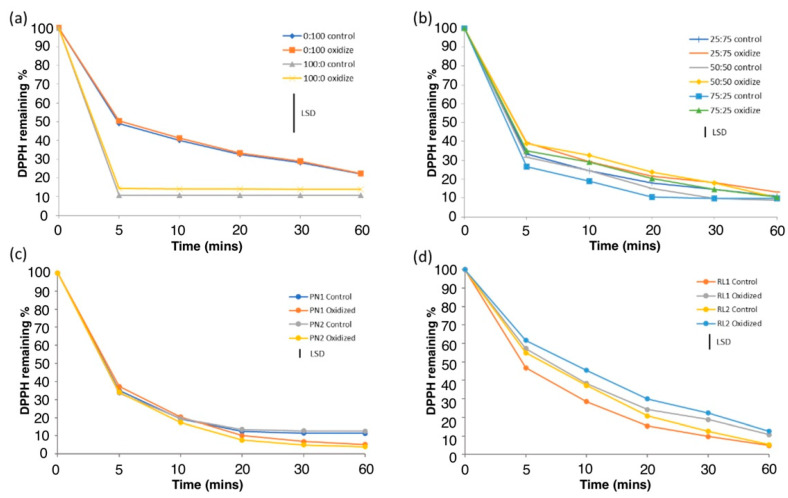
The DPPH remaining percentage of control and oxidized flavonoid model (**a**,**b**) and wine lees (**c**,**d**). The black bar in the figure represents the least significant difference (LSD) of the replicate subsamples samples (n = 3) and measured in triplicate.

The antioxidant activities of the control and oxidized flavonoid model and the wine lees were determined using the DPPH and ORAC assays. The results of the DPPH assay are expressed as percentage of DPPH· remaining throughout a period of 60 min (Figure 3). The composition of the flavonoid model solution had a significant effect on the DPPH· free radical scavenging activity (*p* < 0.05). At 10 min time, the DPPH· remaining percentage was the lowest for Cat:GST 100:0 (10.9 ± 0.2%) and the highest for Cat:GST 0:100 (40.0 ± 1.0%) (Figure 3a). Generally, a higher DPPH· scavenging activity was found in the samples with a higher ratio of catechin. This suggests that monomeric flavan-3-ols have a better DPPH· radical scavenging abilities than tannins.

The DPPH· remaining percentage at 10 min time in the oxidized flavonoid model samples are the lowest in Cat:GST 100:0 (14.2 ± 0.2%) and the highest in Cat:GST 0:100 (41.2 ± 0.2%), respectively. The oxidized samples had a similar trend to that found in the control samples mentioned above. No significant differences between the control and oxidized samples were observed in each Cat:GST mixture (Figure 3b) (*p* > 0.05). This indicated that oxidation did not significantly affect the DPPH· scavenging activity of the phenolics mixture. This suggested that the newly formed products from the H_2_O_2_-dependent oxidation in the presence of HRP have no negative impacts on the DPPH free radicals scavenging ability. During oxidation, flavonol-3-ols subunits can be polymerized by forming the linkages between C_3_, C_4_, C_5_ or C_6_ of one subunit and the C_8_ (or C_6_) of the adjacent subunit [11]. Thus, the hydroxyl groups of these subunits at these positions during oxidation might be replaced by C-C linkage. According to Rice-Evans et al. [34], antioxidant activity of flavonol-3-ols depends on the number of hydroxyl groups. Therefore, the fewer hydroxyl groups as the process of polymerization of the phenolic compounds in the Cat:GST mixture had no significant impacts on antioxidant activity against DPPH free radicals.

The percentages of DPPH· remaining of the control and oxidized wine lees are shown in Figure 3c,d. As the DPPH radical scavenging activity is highly correlated to the TPC of lees [13], thus no significant differences were found between the PN1 and PN2 control samples due to the similar TPC. The RL1 control lees showed lower antioxidant activity than the PN ones. This is because a significantly higher TPC was found in the PN lees than the RL lees (*p* < 0.05) since the vinification technique used in red wine making allows more phenolic extraction into the must than white wine.

A decrease in antioxidant activity was observed in both the oxidized PN and RL samples. However, the difference between the control and oxidized PN samples was not statistically significant (*p* > 0.05). This means that oxidation did not have a significant impact on the antioxidant activity of the PN wine lees against the DPPH free radicals. This observation is consistent with the flavonoid model Cat:GST 0:100. Different observations were found in the RL lees where the DPPH radical scavenging activities of the oxidized RL samples were reduced dramatically after oxidation. The chemical compounds in the lees are complex and may be converted into different products during oxidation in the presence of hydrogen peroxide and HRP. Loss some of phenolic compounds may not impact PN antioxidant activity to the same extent as these lees are rich in phenolic compounds. However, the oxidation of the RL lees has significant impact on antioxidant activity as the TPC in the lees was very low originally.

**Figure 4:**
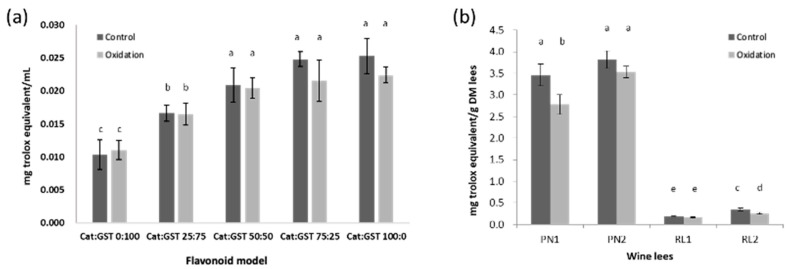
Oxygen radical absorbance capacity (ORAC) (mg Trolox equivalent/mL) of control and oxidized flavonoid model (**a**) and wine lees (**b**). Mean values that do not share a letter (a–e) are significantly different (*p* < 0.05). Error bars are standard deviation of the replicate subsamples samples (n = 3) and measured in triplicate.

The ORAC assay results of the flavonoid model were expressed as milligram TE (Trolox equivalent) per milliliter (Figure 4a). The highest and lowest antioxidant activities (ORAC) of both the control and oxidized flavonoid model were observed in Cat:GST 0:100 (Control, 0.12 ± 0.003 and oxidized, 0.012 ± 0.003 mg TE/mL) and Cat:GST 100:0 (Control: 0.028 ± 0.003 and 0.022 ± 0.001 mg TE/mL), respectively (Figure 4a). The ratio of Cat:GST was found have a significant impact on the ORAC (*p* < 0.05). The ORAC of the control samples was increased when the percentage of catechin was increased. Similar to the DPPH scavenging activity, the ORAC activity in oxidized samples was not significantly different from their corresponding controls. This indicated that oxidation did not significantly affect the AAPH· scavenging activity.

For the wine lees, the impact of oxidation on antioxidant (ORAC) was in agreement with the results of the flavonoid model except for PN1 (Figure 4b). The difference in the ORAC between the control and oxidized PN1 was significant (*p* < 0.05). The decrease in the antioxidant activity of the oxidized lees might indicate that a small number of phenolic compounds may have been converted to new compounds/polymer that have a poorer scavenging ability against AAPH free radicals. The use of LC-MS is recommended for future work to identify those possible unknown compounds and investigate how their structural changes can affect antioxidant activity.

The correlation between mDP and the antioxidant activities of the flavonoid model and PN lees was investigated. No correlation was observed between mDP and the percentage of DPPH remaining (0.09), and a weak negative correlation was observed between mDP and the ORAC assay (−0.22). This indicated that the size of the proanthocyanidin polymers in the current study (most ranged from 10 to 30) has no correlation with the antioxidation assays (DPPH and ORAC). The findings in the current study are consistent with those reported by Zhou et al. [35]. The authors found the antioxidant activities (DPPH and FRAP) of the medicinal mangrove (*Ceriops tagal*) extracts and the mDP values were positively correlated when mDP < 10, but the correlation is lost when the mDP > 10 [35].

### 3.4. Antibacterial and Antifungal Activities of Flavonoid Model Samples

The antimicrobial activities of the flavonoid model samples were determined with the minimum inhibition concentration (MIC) broth microdilution test using standard test microorganisms *E. coli*, *S. aureus* and *C. albicans*. The antimicrobial activities (MIC) are expressed in the mg dry matter (DM) flavonoid model per milliliter. The MIC values in the present study ranged from 0.39 mg/mL to 12.5 mg/mL for the different flavonoid model samples with or without oxidation (Table 6).

Among the control flavonoid model samples, the samples with Cat:GST ratios of 25:75 and 75:25 exhibited high activity against *E. coli*, *S. aureus* and *C. albicans* with MIC of 3.13, 3.13 and 6.25 mg/mL, respectively. The composition of the flavonoid model sample affected the antibacterial activities significantly (*p* < 0.05). Monomeric flavan-3-ols (Cat:GST 100:0) showed the highest antibacterial activity against *E. coli* (1.56 mg/mL), but it was less effective against *S. aureus* and *C. albicans*. In contrast, the antimicrobial activities of polymeric tannins (Cat:GST 0:100) against *E.coli* was not different from the control. Similar results were also found for *C. albicans* where 100% monomeric flavan-3-ols and polymeric tannins had no impact on the microbial growth of the tested organisms. In contrast, *S. aureus* was inhibited by both 100% monomeric flavan-3-ols and polymeric tannins with MIC of 6.25 mg/mL.

The oxidation treatment had a significant impact on the antibacterial activity (*p* < 0.05). It was found that the oxidized flavonoid model extracts were more effective inhibiting the growth of *E.coli* with Cat:GST 0:100 (MIC: 1.56 mg/mL) being the least effective and Cat:GST 100:0 (MIC: 0.39 mg/mL) the most effective. Interestingly, the antibacterial activities of the oxidized Cat:GST 50:50 and 75:25 samples became as effective as Cat:GST 100:0 (Table 6). This may be due to new products formed during the oxidation process, which might have a contribution for a higher antibacterial activity. For example, higher concentrations of procyanidin A were observed in the Cat:GST 50:50, 75:25 and 100:0 samples after oxidation (Table 3), although the increase in the 100:0 was numerical. Possible new compounds formed during enzymatic oxidation are oligomerized epicatechins, theaflavate and bistheaflavate [36,37]. However, it was not possible to identify these compounds in the present study due to their complexity and technical limitations. Oxidation significantly improved the antibacterial activity of the flavonoid model against *S. aureus* (*p* < 0.05), but the activity of the oxidized samples against *C. albicans* was generally unchanged (from 12.5 mg/mL to 6.25 mg/mL).

Among the control samples, PN1, PN2 and RL2 showed the same antibacterial activity (MIC: 5 mg/mL) against *E. coli* and *S. aureus*. The highest antifungal activity was found in the PN1 samples (MIC: 2.5 mg/mL), followed by the PN2 and RL2. The oxidized lees samples had similar antimicrobial activity to the control lees samples. This may indicate the antimicrobial activities of the wine lees were not related to the mDP of flavanol-3-ols. Generally, the results in the current study are contrary to the findings reported for wine where red wines showed higher antimicrobial activities than white wine [36,37,38]. Cheng [2] suggested that the antimicrobial activity may be organism-dependent and is not related to TPC.

The wine lees can be compared with the flavonoid model of Cat:GST 0:100 because the tannin polymers are the major flavan-3-ols in these samples. Among the control samples, all the wine lees showed higher antimicrobial activities particularly against *C. albicans*. This can be attributed to the complexity of the chemical composition of the wine lees and some other compounds that the lees may contain and exhibit microbicide activity. For example, chlorogenic acid, caffeic acid, vanillic acid, syringic acid, quercetin and resveratrol were reported to exhibit antimicrobial activity [38,39,40]. However, the antimicrobial activity of the wine lees showed lower antimicrobial activities than the flavonoid model after oxidation (except against *C. albicans*).

There is limited information regarding the effect of oxidation on antimicrobial activity of wine lees. The current study provided new information regarding correlation between mDP and antimicrobial activities of the flavonoid model. No correlation was observed between mDP and MIC/MBC for all tested microorganisms. In the oxidized samples, the antimicrobial activity may be not only organism-dependent but also compositional dependent. Further investigation will be required to identify and purify the newly formed bioactive compounds and test their antimicrobial activity.

Ampicillin and Amphotericin B are board-spectrum antibiotics in common use today. Ampicillin was about 100 and 4000 times more efficient than the oxidized lees when used against *E.coli* and *S. aureus*, respectively. Furthermore, the antibacterial activity of Amphotericin B is 1000 times higher than the lees against *C. albicans*. Therefore, the activities of the resulted compounds are not of therapeutic activity but may be of use in food applications.

## 4. Conclusions

The oxidation of the phenolics was investigated using both the flavonoid model and wine lees. The flavonoid model provided a simpler matrix for understanding the chemical changes during the oxidation. The effect on TPC, TTC, mDP, antioxidant and antimicrobial activity was determined by comparing oxidized and non-oxidized samples. All investigated samples demonstrated these bioactivities. The oxidation treatment had no significant impacts on the antioxidant activities (DPPH and ORAC) in the model and wine lees except the PN1 lees sample. However, antimicrobial activities of the flavonoid model were found to be improved after the oxidation treatment against *S. aureus* and *E. coli* with a minimum inhibition concentration (MIC) of 1.56 and 0.39 mg/mL. This may indicate that new compounds were formed during the oxidation treatment, and these compounds showed microbicidal activity. LC-MS work is required in the future for the identification of the compounds that are newly formed in the oxidation; further investigation will be required for a better understanding of its antimicrobial activity. It is worth noting that the present study has focused on the phenolics in the lees. Potential other compounds present in the lees that can be involved in the bioactivities of the lees extracts cannot be rolled out. Future studies are encouraged to investigate other potential compounds in wine lees.

## Figures and Tables

**Table 1:** A summary of various catechin (Cat) and grape seed tannin mixtures used to examine the effects of oxidation using horseradish peroxidase and hydrogen peroxide model system. The compounds mixtures were dissolved in ethanol: water: TRIS buffer (pH 7.4) at 2.4: 20 and 19.2 *v*/*v*.

	Catechin (mg)	GST (mg)	Cat:GST
Control	0	200	0:100
	50	150	25:75
	100	100	50:50
	150	50	75:25
	200	0	100:0
Treatment	0	200	0:100
	50	150	25:75
	100	100	50:50
	150	50	75:25
	200	0	100:0

**Table 2:** Weight/Volume ratio (g freeze-dried sample/mL) used in solvent extraction.

Sample	Variety	Weight (g)	Solvent	Volume (mL)	*w*/*v* Ratio
PN1	Pinot noir	0.5000	50% acidified EtOH	5	1:10
PN2	Pinot noir	0.5000	50% acidified EtOH	5	1:10
RL1	Riesling	3.0000	50% acidified EtOH	5	6:10
RL2	Riesling	3.0000	50% acidified EtOH	5	6:10

**Table 3:** Phenolic profile of flavonoid model (control and oxidized) (ppm) quantified by HPLC analysis.

Phenolic Compounds	Cat:GST 0:100		Cat:GST 25:75		Cat:GST 50:50		Cat:GST 75:25		Cat:GST 100:0	
	Control	Oxidized	Control	Oxidized	Control	Oxidized	Control	Oxidized	Control	Oxidized
**Non-flavonoids**										
Gallic acid	11.92 ± 0.14 ^a^	8.90 ± 3.52 ^abc^	9.62 ± 0.36 ^b^	0.84 ± 0.30 ^e^	6.04 ± 0.04 ^c^	1.72 ± 0.32 ^d^	2.46 ± 0.92 ^d^	-	-	-
**Hydroxycinnamic acids**										
Caftaric acid	2.44 ± 0.36 ^bc^	2.28 ± 0.64 ^bc^	2.72 ± 0.14 ^bc^	3.40 ± 0.94 ^b^	3.54 ± 0.64 ^bc^	3.68 ± 1.22 ^bc^	1.68 ± 0.36 ^d^	3.96 ± 0.24 ^a^	6.54 ± 2.82 ^a^	4.30 ± 0.10 ^b^
p-coumaric acid	0.04 ± 0.01 ^a^	0.04 ± 0.02 ^a^	0.06 ± 0.04 ^a^	0.04 ± 0.01 ^a^	0.12 ± 0.08 ^a^	0.10 ± 0.04 ^a^	-	-	0.04 ± 0.02 ^a^	0.02 ± 0.01 ^a^
Hydroxybenzoric acid	-	-	-	-	-	3.32 ± 0.01 ^a^	-	2.30 ± 0.14 ^b^	-	-
**Flavonoids**										
**Flavan-3-ols**										
Catechin	189.36 ± 7.22 ^g^	45.80 ± 2.06 ^h^	530.66 ± 16.78 ^e^	351.08 ± 4.42 ^f^	1085.14 ± 36.32 ^c^	852.28 ± 17.40 ^d^	1571.44 ± 30.86 ^b^	850.30 ± 17.40 ^d^	1879.42 ± 124.30 ^a^	835.40 ± 70.22 ^d^
Epicatechin	66.30 ± 0.58 ^a^	63.20 ± 2.40 ^a^	45.54 ± 0.38 ^b^	2.90 ± 0.18 ^d^	4.00 ± 0.06 ^c^	1.32 ± 0.28 ^e^	2.96 ± 1.20 ^d^	1.62 ± 0.14 ^e^	-	-
Epicatechin gallate	-	-	-	-	-	-	-	-	-	-
Epigallocatechin	0.38 ± 0.01 ^f^	0.36 ± 0.02 ^f^	4.08 ± 0.12 ^d^	2.70 ± 0.04 ^e^	8.34 ± 0.28 ^b^	6.54 ± 0.14 ^c^	12.08 ± 0.24 ^a^	6.54 ± 0.10 ^c^	14.44 ± 0.96 ^a^	6.42 ± 0.54 ^bc^
Procyanidin A *	-	-	7.70 ± 0.18 ^e^	8.14 ± 0.36 ^e^	21.02 ± 0.42 ^d^	47.76 ± 2.16 ^bc^	43.94 ± 0.90 ^c^	59.16 ± 0.82 ^b^	64.66 ± 0.42 ^a^	75.30 ± 5.58 ^a^
Procyanidin B *	9.68 ± 0.24 ^a^	9.54 ± 0.14 ^a^	5.94 ± 0.12 ^b^	-	-	-	-	-	-	-
Procyanidin C *	7.24 ± 0.06 ^a^	6.08 ± 0.14 ^b^	5.18 ± 0.30 ^c^	-	-	-	-	-	-	-

**Note**: all the data are expressed as parts per million (ppm). Means that do not share a letter (a–h) are significantly different (*p* < 0.05). “*”: A, B and C are representing three different kinds of procyanidin identified in samples that are not represent the actual structure. “-“: Not detected.

**Table 4:** Phenolic profile of the Pinot noir and Riesling wine lees (control and oxidized) quantified by LC-MS analysis.

Phenolic Compounds	PN1		PN2		RL1		RL2	
	Control	Oxidized	Control	Oxidized	Control	Oxidized	Control	Oxidized
**Non-flavonoids**								
Gallic acid	1.64 ± 0.01 ^b^	0.07 ± 0.01 ^d^	2.03 ± 0.01 ^a^	0.43 ± 0.18 ^c^	0.13 ± 0.01 ^c^	0.02 ± 0.01 ^e^	0.10 ± 0.04 ^c^	0.07 ± 0.01 ^d^
**Hydroxy** **ci** **nnamic acids**								
Caftaric acid	-	-	-	-	-	-	-	-
Cinnamic acid	-	-	-	-	0.05 ± 0.01 ^a^	0.05 ± 0.01 ^a^	-	-
p-coumaric acid	-	-	-	-	-	-	-	-
Hydroxybenzoric acid	-	-	-	-	-	-	-	-
**Stillbenoids**								
Resveratrol	0.42 ± 0.01 ^b^	0.34 ± 0.01 ^c^	0.62 ± 0.01 ^a^	0.40 ± 0.01 ^b^	0.10 ± 0.01 ^e^	0.08 ± 0.01 ^e^	0.29 ± 0.01 ^d^	0.41 ± 0.01 ^b^
**Flavonoids**								
**Flavan-3-ols**								
Catechin	3.80 ± 1.50 ^c^	0.11 ± 0.04 ^g^	3.63 ± 0.09 ^c^	0.69 ± 0.04 ^e^	0.92 ± 0.06 ^d^	0.22 ± 0.02 ^f^	16.34 ± 0.49 ^a^	8.45 ± 1.48 ^b^
Epicatechin	1.70 ± 0.05 ^b^	0.28 ± 0.05 ^d^	2.33 ± 0.08 ^a^	0.48 ± 0.03 ^c^	2.43 ± 0.14 ^a^	0.40 ± 0.05 ^c^	0.17 ± 0.04 ^e^	0.19 ± 0.01 ^e^
Epicatechin gallate	0.76 ± 0.02 ^c^	0.29 ± 0.02 ^d^	0.83 ± 0.02 ^b^	0.29 ± 0.01 ^d^	0.32 ± 0.18 ^d^	0.13 ± 0.05 ^e^	1.57 ± 0.93 ^a^	2.59 ± 0.08 ^a^
Epigallocatechin	0.02 ± 0.01 ^c^	-	0.03 ± 0.01 ^c^	-	-	-	0.13 ± 0.01 ^a^	0.06 ± 0.01 ^b^
Procyanidin A	2.39 ± 0.01 ^d^	2.55 ± 0.14 ^d^	2.14 ± 0.09 ^e^	2.97 ± 0.15 ^c^	0.48 ± 0.08 ^b^	0.40 ± 0.02 ^b^	5.77 ± 1.27 ^a^	4.46 ± 0.14 ^a^
Procyanidin B	1.65 ± 0.02 ^a^	1.16 ± 0.04 ^a^	1.43 ± 0.03 ^a^	1.39 ± 0.02 ^a^	-	-	2.55 ± 1.92 ^a^	0.05 ± 0.04 ^b^
Procyanidin C	1.79 ± 0.17 ^b^	4.22 ± 0.10 ^a^	1.61 ± 0.02 ^b^	3.88 ± 0.23 ^a^	-	-	-	-
**Anthocyanins**								
Cyanidin-3-glucoside	-	-	-	-	-	-	-	-
Delphinidin-3-glucoside	-	-	-	-	-	-	-	-
Malvidin-3-glucoside	0.02 ± 0.01 ^b^	-	0.05 ± 0.01 ^a^	-	-	-	-	-
Peonidin-3-glucoside	-	-	-	-	-	-	-	-
Petunidin-3-glucoside	-	-	-	-	-	-	-	-
**Flavanols**								
Quercetin	1.80 ± 0.05 ^b^	0.97 ± 0.01 ^d^	2.38 ± 0.01 ^a^	1.50 ± 0.09 ^c^	0.77 ± 0.04 ^e^	0.21 ± 0.01 ^f^	1.10 ± 0.11 ^d^	1.26 ± 0.01 ^d^
Quercetin methyl-glucoside	1.16 ± 0.01 ^a^	1.03 ± 0.03 ^b^	0.79 ± 0.03 ^c^	0.92 ± 0.02 ^b^	0.42 ± 0.01 ^d^	0.20 ± 0.01 ^e^	1.01 ± 0.15 ^ab^	0.91 ± 0.01 ^b^

**Note**: all the data are expressed mg/g DM lees. Means that do not share a letter (a–g) are significantly different (*p* < 0.05). “-“: Not detected.

**Table 5:** The summarized table of mean degree of polymerization (mDP) and percentage of monomers in the breakdown of the phenolic in polymer fractions in the Pinot noir wine lees.

			Terminal Units (%)				Extension Units (%)			
Sample	mDP	Treatment	C	EC	ECG	EGC	C	EC	ECG	EGC
PN1	10.7 ± 0.5 ^a^	Control	64.40	31.59	4.01	0.00	6.89	59.54	3.09	30.48
PN2	6.7 ± 0.4 ^c^	Control	58.63	37.52	3.85	0.00	7.21	58.10	3.00	31.69
PN1	10.6 ± 0.9 ^a^	Oxidized	54.37	37.82	7.81	0.00	12.03	44.22	2.36	41.38
PN2	6.8 ± 1.1 ^bc^	Oxidized	55.30	39.81	4.89	0.00	12.22	41.70	2.10	43.98

C: Catechin; EC: Epicatechin; ECG: Epicatechin Gallate; EGC: Epigallocatechin; PG: Phloroglucinol adduct. Means that do not share a letter (a–c) within significantly different (*p* < 0.05).

**Table 6:** Minimum inhibitory concentration (MIC) and minimum bactericidal concentration (MBC) values of oxidized and non-oxidized (control) flavonoid model (Cat:GST at 0:100, 25:75, 50:50, 75:25 and 100:0 ratios) and Pinot noir (PN1 and PN2) and Riesling (RL1 and RL2) wine lees.

		Antibacterial/Antifungal Activity (MIC50) mg/mL					
		*S. aureus* ^1^		*E. coli* ^1^		*C. albicans* ^2^	
Sample		(MIC) mg/mL	(MBC) mg/mL	(MIC) mg/mL	(MBC) mg/mL	(MIC) mg/mL	(MBC) mg/mL
Control	0:100	6.25	6.25	12.50 *	12.50 *	12.50 *	12.50 *
	25:75	3.13	3.13	3.13	3.13	**6.25**	12.50 *
	50:50	12.50 *	12.50	6.25	6.25	**6.25**	12.50 *
	75:25	3.13	3.13	3.13	3.13	**6.25**	12.50 *
	100:0	6.25	6.25	1.56	3.13	12.5 *	12.50 *
Oxidized	0:100	3.13	**3.13**	1.56	3.13	12.5 *	12.50 *
	25:75	1.56	**3.13**	0.78	0.78	**6.25**	**6.25**
	50:50	3.13	**3.13**	0.39	0.78	**6.25**	12.50 *
	75:25	**1.56**	**3.13**	0.39	0.78	**6.25**	**6.25**
	100:0	3.13	**3.13**	**0.39**	**0.39**	**6.25**	12.50 *
Control	PN1	**5.00**	10.00	**5.00**	10.00	**2.50**	**5.00**
	PN2	**5.00**	10.00	**5.00**	**5.00**	5.00	10.00
	RL1	10.00	20.00	10.00	20.00	10.00	20.00
	RL2	**5.00**	10.00	**5.00**	10.00	5.00	10.00
Oxidized	PN1	**5.00**	10.00	**5.00**	10.00	**2.50**	10.00
	PN2	**5.00**	10.00	**5.00**	10.00	5.00	10.00
	RL1	10.00	20.00	10.00	20.00	10.00	20.00
	RL2	**5.00**	**5.00**	**5.00**	10.00	5.00	10.00
		**Antibacterial/antifungal activity (MIC)** **µ** **g/mL**					
**Antimicrobial Agent**		*S. aureus* ^1^		*E. coli* ^1^		*C. albicans* ^2^	
Ampicillin		**0.781**		**3.125**			
Amphotericin B						**6.25**	

^1^ MIC values after 24 h incubation with bacteria. ^2^ MIC values after 48 h incubation with fungi. * = not different from DMSO (the solvent). **Bold type** indicates the highest antibacterial and antifungal activity (MIC50) of flavonoid model solution or wine lees sample in each column.

**Figure A1:**
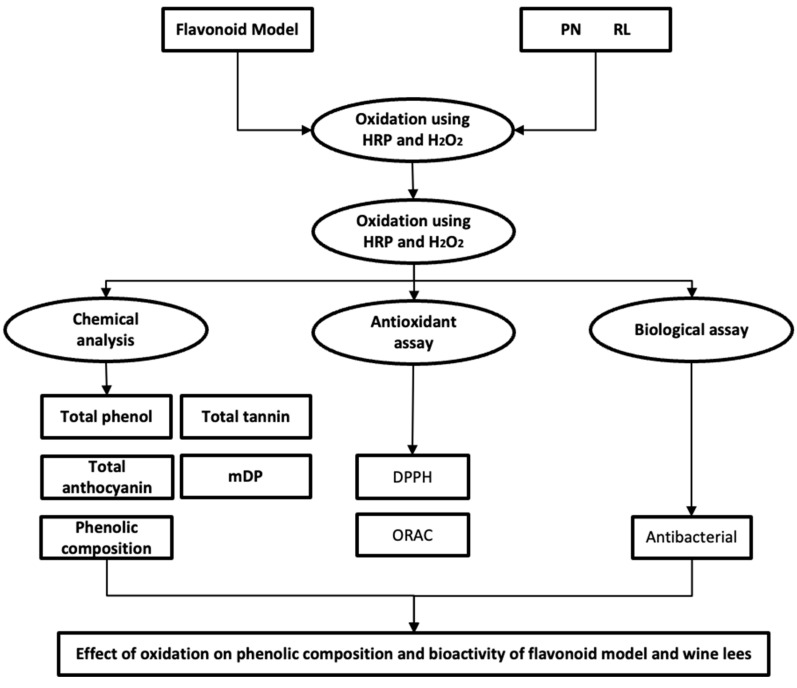
The experimental design.

## Data Availability

The data presented in this study are available in the article.

## Appendix A

**Table A1:** Summary of vinification used for PN and RL winemaking.

Sample	Juice Type	Vessel	Yeast (N/C)	Fermentation Temp (°C)	Maceration	Vinification	Lees Collection
PN1	FR	PV	N	34	Pre and Post	PO, P	Pre-MLF
PN2	FR	PV	N	35	Pre and Post	PO, P	Pre-MLF
RL1	-	Tank	C	17	-	-	Post-ALC
RL2	-	Tank	C	15	-	-	Post-ALC

Note: FR: Free run; PV: portable vessel; N/C: Natural/Commercial Yeast; Pre- or Post-: Pre-fermentation maceration or Post-fermentation maceration; PO: Pump over: P: Plunging; Pre-MLF: Pre-Malolactic fermentation; Post-ALC: Post alcoholic fermentation.
